# *CYP19A1* promoter methylation in saliva associated with milestones of pubertal timing in urban girls

**DOI:** 10.1186/1471-2431-14-78

**Published:** 2014-03-20

**Authors:** Theresa Ryan Stueve, Mary S Wolff, Ashley Pajak, Susan L Teitelbaum, Jia Chen

**Affiliations:** 1 Department of Preventive Medicine, Icahn School of Medicine at Mount Sinai, New York, NY, USA; 2 Department of Preventive Medicine, Keck School of Medicine, University of Southern California, Los Angeles, CA, USA

**Keywords:** Aromatase, Methylation, Obesity, PPAR, Puberty

## Abstract

**Background:**

Childhood obesity and early puberty are intermediate risk factors for later metabolic and reproductive disorders including diabetes, polycystic ovarian syndrome (PCOS), and breast cancer. Atypical methylation patterns in genes related to hormone and adipose metabolism, such as *CYP19A1* (*aromatase*) and *PPARG* (*peroxisome proliferator-activated receptor gamma*), are associated with alterations in gene expression which may contribute to pathogenesis of these diseases. If present in early life, it is conceivable similar methylation aberrations may result in hormone perturbations that alter pubertal timing.

**Methods:**

We used Cox proportional hazard models to investigate whether promoter methylation of *CYP19A1* and *PPARG*, independently or in concert with body weight, was associated with age at breast (B2) or pubic hair development (PH2) when assayed in saliva DNA collected from a cohort of New York City, Black and Hispanic girls (N = 130) enrolled in a study of pubertal timing between 6–8 years of age.

**Results:**

An inverse association between *CYP19A1* methylation and risk of early PH2 was suggested (HR = 0.95, 95% CI = 0.90-1.00, p = 0.05). *CYP19A1* methylation also appeared to modify risk of early B2 associated with body weight. Specifically, compared to normal weight girls with ‘high’ *CYP19A1* methylation, significantly increased risk of early B2 was observed in overweight girls with ‘low’ but not ‘high’ *CYP19A1* methylation (HR = 2.15; 95% CI = 1.23- 3.76). However, in formal tests for effect modification, the interaction between body weight and methylation did not reach statistical significance (p for interaction = 0.085). *PPARG* methylation was not significantly associated with PH2 or B2.

**Conclusions:**

Though limited by sample size, our findings suggest methylation of *CYP19A1*, a critical gene in estrogen biosynthesis*,* may influence timing of breast development in overweight girls. Consistent with emerging reports, these data support the notion that epigenetic marks in surrogate tissues may improve risk prediction when added to standard plasma and anthropometric indicators, and warrant further study.

## Background

Emerging evidence indicates modifiable lifestyle factors and time-stable epigenetic determinants influence childhood obesity [1,2] and timing of puberty [3]. Childhood obesity and early puberty are risk factors for several metabolic and reproductive disorders, including: early menarche [4], adult obesity [5], diabetes [6], polycystic ovarian syndrome (PCOS) [7], and breast cancer [8]. PCOS and its common childhood antecedent, premature pubarche, are associated with hyperinsulinemia and androgen excess in overweight females [7,9]. Similarly, plasma leptin [10] and local adiposal estrogen [11] are associated with breast tumor growth in obese postmenopausal cases, and are hypothesized to drive early breast development and menarche in overweight girls [12-15]. Given that the ovaries are largely quiescent pre-menarche and post-menopause, it is conceivable biomarkers that link adiposity to pubertal timing may inform risk of androgen and estrogen-related disease development throughout the life course. In this exploratory pilot study we asked whether promoter methylation marks in *PPARG* or *CYP19A1*, two genes that connect energy balance to lifetime estrogen exposure, are associated with pubertal development in girls.

The transcription factor PPARγ is a master regulator of adipose differentiation and endocrine function. Human and animal data link *PPARG* hypermethylation to reduced PPARγ expression that is observed in cases of diabetes [16], breast cancer [17], and hyperandrogenic PCOS [9]. Because PCOS and early pubarche share hyperinsulemia and androgen excess as common clinical features, it is conceivable *PPARG* methylation aberrations may alter timing of pubarche if present in early development [9].

Aromatase (product of the *CYP19A1* gene) catalyzes all bodily estrogen biosynthesis *via* aromatization of androgen precursors, and inhibitors of this enzyme are highly effective therapies for estrogen-sensitive cancers of the breast [18]. Aromatase expression varies across tissues and individuals owing to differential activation and repression of several tissue-specific gene promoters. Each of these promoters regulates a unique untranslated ‘first exon’ (‘exons I’) that is spliced to the common coding exons II-X and can be mapped back to its promoters for purposes of deducing what factors drive transcription in a particular tissue [19]. In healthy adipose, *CYP19A1* is expressed in fibroblasts primarily from activation of a distal glucocorticoid-regulated promoter termed ‘pI.4’, and is lost in the path of adipocyte differentiation driven by PPAR*γ*[20]. In malignant breast biopsies, *CYP19A1* is overexpressed 3–4 fold in tumor and proximal adipose tissue from several tissue-specific promoters [21], but primarily from two largely ‘gonad-specific’ cAMP-responsive promoters termed “*pII/I.3*” [22] that are activated by cancer-associated transcription factors PPARγ suppresses in healthy breast [17,23]. Though this phenomenon of increased tissue-specific promoter usage with preference for gonadal promoters (termed ‘promoter switching’) is well-documented in the breast cancer literature [19,24], its timing and extent of tissue distribution related to intermediate risk indicators is less understood. Demura and Bulun [25] recently described hypomethylation of a CpG dinucleotide in the ‘cAMP-response element-like sequence’ (CLS) of *pII/I.3*, which they detected in aromatase-overexpressing fibroblasts derived from a skin-punch biopsy of a healthy patient. In light of the aggregate of findings regarding CYP19A1 misexpression from gonadal promoters in breast cancer cases, Demura and Bulun postulated *CYP19A1 pII/I.3* hypomethyaltion may contribute to the phenomenon of ‘promoter switching’ and inter-individual variability in lifetime estrogen exposure.

In the present study we sought to determine whether methylation of this *CYP19A1 pII/I.3* locus [25] or the average of five CpG dinucleotides in a differentially methylated region of the *PPARG* promoter [9,16,26] was associated with timing of pubic hair (PH2) or breast development (B2) in a cohort of New York City, Black and Hispanic girls who were enrolled in a study of pubertal timing between 6–8 years of age.

## Methods

### Study population

#### Growing up healthy

Prospective cohort study and part of the Puberty Studies of the Breast Cancer and Environment Research Program (BCERP) [27]. The overarching goal of this longitudinal investigation is to identify genetic and environmental risk factors related to altered timing of puberty onset in girls. Girls, 6 to 8 years of age, from East Harlem schools, community health centers, and the Mount Sinai Pediatric clinic were recruited for this study between 2004–2007, as previously reported [28]. Consent was obtained from parents or guardians and child assent was independently verified; the study was approved by the Institution Review Board at Mount Sinai. Eligibility included age, female sex, no underlying endocrine medical conditions, and self-identified Black or Hispanic race/ethnicity. A total of 416 girls were enrolled in the study at baseline; we restricted our analysis to the 130 who had whole saliva collected. The distribution of major demographic and physiological variables, including race, caregiver’s education, BMI, breast stage and pubic stage, showed no significant difference between those who donated saliva samples and those who did not (data not shown).

### Demographic and anthropometric data collection

Uniformly trained interviewers conducted annual in-person interviews and standardized anthropometric measurements. Annual pubertal stage assessments were performed by physicians or nurse practitioners according to BCERP consortium-standardized protocols; the principal endpoints were age at first pubic hair and breast development as described in detail previously [27]. A structured questionnaire was administered to the girl’s parent or guardian in either English or Spanish. Information ascertained through the questionnaire included medical history and demographic variables. Body mass index (BMI) was calculated as weight (in kg) divided by height (in cm)-squared. We classified girls as ‘overweight’ according to Centers for Disease Control (CDC) and Prevention criteria, where overweight girls had a BMI at or above the 85^th^ percentile of their age and sex-specific BMI distribution. Age at B2 was defined as the midpoint between the age at the last visit where the girl was staged B1 with no prior staging greater than B1 and the age at the first visit where the girl was staged B2 with no subsequent staging less than B2. Girls who entered the study at B2 were assigned age at B2 as six months prior, and girls with a breast stage less than B2 at their last visit were right censored. Age at PH2 was assigned in the same manner.

### Saliva DNA collection and processing

Interviewers instructed study participants to deposit saliva in pre-barcoded 2 ml Oragene DNA Self-Collection Kit tubes (DNA Genotek, Canada; REF OG-100) according to the manufacturer’s instructions. Barcoded vials were logged in our database by scanning upon receipt in the laboratory. DNA was extracted from whole saliva collected in Oragene tubes (DNA Genotek) with the ITprep kit (DNA Genotek) according to the manufacturer’s instructions.

### Methylation assessment by pyrosequencing

Genomic DNA was bisulfite-converted with the Epitect DNA kit (Qiagen, Carslbad, CA). Pyrosequencing was performed on *CYP19A1* and *PPARG* PCR products amplified from bisulfite-treated DNA with the Pyromark PCR kit (Qiagen) and the primers listed in Additional file 1. Cycling conditions were 95°C for 15 minutes followed by: 45 cycles of 94°C for 30 seconds; annealing temperature for 30 seconds; 72°C for 30 seconds; with a final extension step of 72°C for 10 minutes. PCR products were sequenced using the Pyromark Q24 system and kit (Qiagen). Percent methylation for each region of interest was quantified using Pyromark Q24 software version 1.0.1 (Qiagen). Genomic coordinates for the promoter regions amplified are included in Additional file 1; coordinates were obtained from the UCSC Genome Browser (http://genome.ucsc.edu), human assembly: February 2009, GRCh37/hg19). Laboratory personnel performing DNA methylation analysis were blinded to subject information.

### Statistical analysis

We examined relationships among methylation and study characteristics with parametric (t-tests) and non-parametric (Mann-Whitey U tests) statistics and multivariate linear regression. Cox proportional hazard models were used to identify associations between DNA methylation and age at PH2 or B2. Interaction was examined by including a group variable that was constructed by pairing the dichotomized methylation (below vs. greater than or equal to their medians) and dichotomized body size (normal weight vs. overweight). All models were adjusted for Hispanic ethnicity, Black race, and caregiver education level. All analyses were performed using SAS (version 9.1.3 for PC; SAS Institute Inc., Cary, NC).

## Results

### Study population demographics according to *CYP19A1* and *PPARG* methylation

Study subjects (N = 130) were Black or Hispanic girls living in the East Harlem neighborhood of New York City. Girls were recruited in local clinics and community centers between 2004–2007, and were 6 (72 months) to 8.9 (107 months) years old with a mean age of 7.5 years (89.5 months) at time of enrollment. Based on CDC criteria, 39.2% of our study subjects were considered overweight (≥ 85^th^ percentile of their age and sex-specific BMI distribution) and 25.4% were considered obese (≥ 95^th^ percentile). Of the study subjects’ primary caregivers, 59% had completed high school. Among the 130 whole saliva samples collected, 5 failed the pyrosequencing assay for *CYP19A1* and 1 for *PPARG*, leaving 125 and 129 samples, respectively, with methylation data. *CYP19A1* methylation values ranged from 77% to 95% (mean: 88.8% ± 3.5% SD). *PPARG* methylation ranged from 5.6% to 19% (mean: 10.4% ± 2.1% SD). Associations between methylation levels and key demographic variables are summarized in Table 1. No significant differences were observed with respect to race, ethnicity, BMI percentile, or caregiver’s education level.

**Table 1:** **Study population demographics according to *CYP19A1 *** **and *PPARG *** **methylation (%)**

	***CYP19A1 *** **methylation (%)**			***PPARG *** **methylation (%)**		
**Characteristic**	**N**	**Mean (SD)**	**p**	**N**	**Mean (SD)**	**p**
**Overall**	125	88.84 (3.45)	-	129	10.45 (2.14)	-
**Child ethnicity**						
Hispanic*	100	89.06 (3.39)	0.75**	102	10.51 (2.18)	0.28**
***Not hispanic***	25	87.96 (3.59)		27	10.21 (2.00)	
**Child race**						
Black*	44	88.48 (3.39)	0.39	45	10.45 (2.36)	0.98
***Not black***	81	89.03 (3.48)		84	10.44 (2.02)	
**Caregiver education**						
***≤High school***	75	89.12 (3.58)	0.34	78	10.37 (2.17)	0.93
***>High school***	45	88.49 (3.31)		46	10.40 (2.14)	
**Baseline BMI**						
***<85***^***th***^	76	88.80 (3.53)	0.88	79	10.47 (2.06)	0.86
***≥85***^***th***^	49	88.89 (3.37)		50	10.4 (2.28)	

*includes 19 individuals who are self- identified as Black Hispanics.

**p for the nonparametric Mann Whitney U test.

### Gene methylation related to milestones of pubertal development

We investigated whether methylation of *CYP19A1* or *PPARG* was related to age at B2 or PH2 using Cox Proportion Hazards Models (Table 2). For PH2, we observed an inverse association with *CYP19A1* methylation in unadjusted models; for a one percent increase in *CYP19A1* methylation, girls were 5% more likely to be older at PH2 (HR = 0.95; 95% CI = 0.90-0.99). This association was attenuated in models adjusted for ethnicity, BMI percentile, and caregiver’s education (HR = 0.95, 95% CI = 0.90-1.00). Conversely, no significant associations between age at B2 and *CYP19A1* methylation were observed. In addition, no significant associations among *PPARG* methylation and PH2 or B2 were observed.

**Table 2:** **Associations between *CYP19A1 *** **and *PPARG *** **methylation (%) and age at pubertal development.**

	**Unadjusted model**			Adjusted model*		
	**N**	**HR (95% CI)**	**p value**	**N**	**HR (95% CI)**	**p value**
**Pubic Hair (PH2) development**						
***CYP19A1***	125	0.95 (0.90 - 0.99)	0.04	120	0.95 (0.90 - 1.00)	0.05
***PPARG***	129	0.99 (0.90 - 1.08)	0.75	124	0.96 (0.87 - 1.05)	0.37
**Breast development (B2)**						
***CYP19A1***	125	0.97 (0.92 - 1.02)	0.17	120	0.96 (0.91 - 1.01)	0.11
***PPARG***	129	1.04 (0.96 - 1.13)	0.32	124	1.03 (0.94 - 1.12)	0.54

*Cox proportional hazard models were adjusted for child race/ethnicity, BMI percentile, and caregiver education. Education information was missing from 5 subjects. 95% CI = 95% confidence interval.

### Effect of body size modified by gene methylation

Obesity is one of the strongest predictors of pubertal onset [29]. Therefore we next sought to determine whether gene methylation modifies the relationship between BMI and age at PH2 and B2. We created ‘normal weight’ and ‘overweight’ categories of body size (below and greater than or equal to the 85^th^ percentile for age and sex-specific BMI distribution), and ‘high’ and ‘low’ methylation (above and below the median). As shown in Table 3, compared to normal weight girls with high *CYP19A1* methylation (low risk referent), risk of earlier breast development was greater among overweight girls with low *CYP19A1* methylation (HR = 2.15, 95% CI = 1.23 - 3.76). This BMI-methylation interaction reached borderline significance in formal tests for effect modification (p for interaction = 0.085). A similar effect was observed for *CYP19A1* methylation and age at PH2, although the interaction did not reach statistical significance (p for interaction = 0.21). Lastly, no significant interactions between BMI and *PPARG* methylation in relation to PH2 or B2 were detected.

**Table 3:** Interaction of body size and relative gene methylation in predicting age at pubertal development

	**Pubic hair (PH2)**		**Breast (B2)**	
	**N**	**HR (95% CI)**	**N**	**HR (95% CI)**
***CYP19A1 *** **(N = 120)**				
**Normal weight, high methylation**	37	1.00 (referent)	37	(referent)
**Normal weight, low methylation**	39	1.04 (0.63, 1.7)	39	0.82 (0.50, 1.33)
**Overweight, high methylation**	25	1.05 (0.61, 1.81)	25	1.32 (0.78, 2.23)
**Overweight, low methylation**	24	1.80 (1.04, 3.12)	24	2.15 (1.23, 3.76)
**p for interaction**		0.21		0.085
***PPARG *** **(N = 124)**				
**Normal weight, low methylation**	35	1.00 (referent)	35	1.00 (referent)
**Normal weight, high methylation**	44	1.10 (0.65, 1.85)	44	1.33 (0.81, 2.18)
**Overweight, low methylation**	29	1.42 (0.83, 2.42)	29	1.84 (1.08, 3.13)
**Overweight, high methylation**	21	1.21 (0.67, 2.18)	21	1.94 (1.08, 3.49)
**p for interaction**		0.52		0.55

Overweight was defined as BMI at or above the 85^th^ percentile for age. ‘Low’ and ‘high’ methylation were defined as below or above the median level of % methylation for each gene locus (89% for *CYP19A1*, 10.4% for *PPARG)*. Models were adjusted for child race/ethnicity, and caregiver education (< vs. ≥ high school).

## Discussion

Early breast and pubic hair development have been associated with disordered leptin, insulin, and IGF-1 profiles in overweight girls in numerous studies [30]. Perturbations in estrogens and androgens, critical drivers of breast and pubic hair development, remain clinically more challenging to detect [31]. Given national trends, there is great motivation to identify biomarkers that add value to current plasma and anthropometric measures used in predicting puberty onset [32]. In this exploratory study we aimed to ascertain whether salivary methylation of the *CYP19A1* and *PPARG* promoters was related to age at breast or pubic hair development in girls, both independently and in concert with body size. In light of the current literature, we anticipated overweight girls with *CYP19A1* hypomethylation and *PPARG* hypermethylation might be predisposed to early breast development [33-35], and those with *PPARG* hypermethylation to early pubic hair development [9,16].

Our main observations were that relative hypomethylation of a CpG in the gonadal *CYP19A1* promoter termed *“pII* “ was associated with earlier age at B2 among overweight girls only (Table 3), and with earlier age at PH2 independent of body size (Table 2). While only correlative and based on a relatively small number of samples, our B2 findings are supported by a case report authored by Demura and Bulun [25], which describes hypomethylation of *pI.3/II* in CYP19A1-overexpressing fibroblasts relative to CYP19A1-quiescent fibroblasts derived from punch biopsies of four healthy subjects. In their report, CYP19A1 activity was robustly induced in the former upon cAMP stimulation, while fibroblasts from the other three subjects were cAMP-refractory. Further investigation revealed CpG dinuleotides within and proximal to the CLS (CRE-like sequence) of gonadal *pI.3/II* were relatively hypomethylated in cAMP-responsive CYP19A1-overexpressing fibroblasts, and were relatively hypermethylated in non-responsive fibroblasts. These results support the hypothesis that *CYP19A1* hypomethylation may be an early ‘permissive’ event, which renders one susceptible to subsequent intrinsic/extrinsic transcriptional activators of *CYP19A1,* and concomitant local or systemic estrogen excess. Such a ‘two-hit’ mechanism of derepression (hypomethylation) and activation (e.g., obesity-related cytokines) may also explain why *CYP19A1* hypomethylation was associated with early B2 in overweight, but not normal weight girls in the present study.

Aromatase catalyzes estrogen biosynthesis from androgen precursors. Elevated androgen, insulin, and IGF-1 signaling are widely accepted co-determinants of early pubarche in overweight girls [7,30,36]. Thus, our finding that *CYP19A1* hypomethylation (theoretical increase in expression) was related to earlier age at PH2, independent of BMI (Table 2), was unanticipated. While intriguing, the statistical significance of this association was attenuated after adjustment for covariates, and we can only speculate as to its implications without further study.

We assessed methylation of the −383 to −281 bp region of the *PPARG* promoter as PPARγ suppresses CYP19A1 expression in breast tissues in culture [20,33], and relative hypermethylation of this region has been associated with reduced PPARγ expression in hyperandrogenic PCOS [9] and diabetes models [16]. Though we detected no statistically significant effects related to *PPARG* methylation in the present study, puberty-associated methylation patterns may exist in genes for PPARγ co-factors, effectors, or downstream targets in salivary or other surrogate tissue DNA. Indeed, methylation biomarkers of childhood adiposity and maternal BMI have been described in *RXRA* and *PPARGC1A* when assayed in umbilical tissue [2,37].

This exploratory investigation has several limitations regarding generalizability, including but not limited to: small sample size, lack of perceived stress assessments, use of candidate genes, and DNA derived from whole saliva samples collected only from Black and Hispanic girls. We describe salivary *CYP19A1* hypomethylation not as a ‘causal’ event, but merely as a ‘surrogate biomarker’ that with further study may have utility in predicting risk of premature breast development in overweight girls. Specifically, the CpG we describe is contained in a critical transcription factor binding site (CLS), located in a strong *CYP19A1* gonadal promoter termed ‘*pII*’, which is activated by the ubiquitous pleiotropic second messenger cAMP in the follicular phase of the menstrual cycle [38]. DNA methylation is highly tissue-specific, and CYP19A1 is not likely expressed in buccal epithelial cells from gonadal *pII* to any significant degree. However, weighing these considerations together supports the notion that hypomethylation of *CYP19A1 pII* in whole saliva- a tissue in which expression from gonadal *pII* is likely silenced, may in fact represent a methylation aberration, possibly established early in life. Such a mark could be deemed a ‘surrogate’ aberration if it portends risk generalizable to more functional tissues with niche transcriptional machinery requisite to affect CYP19A1 expression changes that promote disease, as has been extensively reported for *pII* and estrogen-related disorders [22,24].

Our findings are only suggestive and only extend to saliva samples we collected from Black and Hispanic girls. Procurement of effector/target tissues (e.g. adipose/breast) to investigate the validity of salivary *pII* methylation as a risk surrogate in a statistically robust manner in more diverse pediatric populations is precluded by ethical, logistical, and economic considerations. However, animal and cell line co-culture models designed to capture adipose and developing breast tissue interactions are emerging [39,40], and it will be interesting to follow developments that functionally characterize the complex biological and environmental interactions that orchestrate epigenetic factors related to thelarche and pubarche onset.

## Conclusions

Consistent with emerging human and animal studies, our findings suggest methylation of *CYP19A1* may influence timing of breast development in overweight girls. These data warrant further investigation, and support the notion that epigenetic biomarkers may one day add value to current plasma and anthropometric measures used in predicting timing of puberty onset.

## Abbreviations

B2: Breast development Tanner stage 2; BCERC: Breast Cancer and Environment Research Center; BMI: Body mass index; bp: Base pair; cAMP: Cyclic adenosine monophosphate; CI: Confidence interval; CLS: CRE-like sequence; CRE: cAMP-response element; CREB: CRE binding protein; CYP19A1: *CYP19A1* gene (*aromatase*); HR: Hazard ratio; IGF: Insulin-like growth factor; kB: Kilobase; NCI: National Cancer Institute; NCRR: National Center for Research Resources; NIEHS: National Institutes of Environmental Health Sciences; PCOS: Polycystic ovarian syndrome; PH2: Pubic hair development Tanner stage 2; stages 2 & higher collapsed in this study; PPARG: Gene encoding peroxisome proliferator-activated receptor gamma; SD: Standard deviation; tSS: Trascription start site.

## Competing interests

All authors declare that they have no competing interests.

## Authors’ contributions

MSW and SLT are the co-PIs of the parent study. TRS and JC contributed to the design of this sub-study. TRS performed pyrosequencing analysis, all related laboratory procedures, and assumes the primary role of composing the manuscript with the supervision of JC. SLT and AP performed statistical analysis. All authors were responsible for critical revision of the manuscript and approved the final copy.

## Pre-publication history

The pre-publication history for this paper can be accessed here:

http://www.biomedcentral.com/1471-2431/14/78/prepub

## Supplementary Material

Additional file 1 Bisulfite pyrosequencing primer sets. Click here for file
