# Supplementary material for: CYP19A1 promoter methylation in saliva associated with milestones of pubertal timing in urban girls
Source: BMC Pediatr. 2014 Mar 20;14:78. doi: 10.1186/1471-2431-14-78 (PMC4000125; doi:10.1186/1471-2431-14-78)
Supplement: Additional file 1 — Bisulfite pyrosequencing primer sets. [file 1471-2431-14-78-S1.docx]

# Additional Files

## Additional File 1: Bisulfite pyrosequencing primer sets

| Pyrosequencing primers (Coordinates* from UCSC Feb. 2009 Assembly; *indicates primer with 5’-biotin label) | | | | | |
| --- | --- | --- | --- | --- | --- |
| **(Chromosomal coordinates for region amplified)** | **Forward Primer 5’-3’** | **Reverse Primer 5’-3’** | **Annealing Temperature** | **Sequencing Primer** | **Product Size** |
| *CYP19A1*  chr15:51,535,315-51,535,460 | *AATTTGGTTTTTAATTGGGAATGTA | CCAAAAAAAATCTTTTAACTTAAATTACA | 48 | CTTAAATAAATAAAATAA | 144 |
| *PPARG*  chr3:12,328,967-12,329,068 | TGTTATTGGAAAGAATATTTTGGGAAGA | *TCTAAAAAAACCCAAATATAAAACTCCC | 51 | TGGAAAGAATATTTTGGG | 102 |

*biotinylated primer
